# *Caenorhabditis elegans* Parkin: Regulators of its abundance and role in autophagy-lysosomal dynamics

**DOI:** 10.12688/openreseurope.14235.1

**Published:** 2022-02-18

**Authors:** Roman Vozdek, Bingying Wang, Kathy H. Li, Peter P. Pramstaller, Andrew A. Hicks, Dengke K. Ma

**Affiliations:** 1 Institute for Biomedicine, Eurac Research, Affiliated institute of the University of Lübeck, Bolzano, 39100, Italy; 2 Cardiovascular Research Institute and Department of Physiology, University of California San Francisco, San Francisco, CA, 94158, USA; 3 Department of Pharmaceutical Chemistry, University of California San Francisco, San Francisco, CA, 94158, USA

**Keywords:** Parkinson’s disease, Parkin, Synuclein, genetic screen, RNA interference, autophagy, C. elegans

## Abstract

**Background:** Parkin, which when mutated leads to early-onset Parkinson’s disease, acts as an E3 ubiquitin ligase. How Parkin is regulated for selective protein and organelle targeting is not well understood. Here, we used protein interactor and genetic screens in *Caenorhabditis elegans* ( *C. elegans)* to identify new regulators of Parkin abundance and showed their impact on autophagy-lysosomal dynamics and alpha-Synuclein processing.

**Methods:** We generated a transgene encoding mCherry-tagged *C. elegans* Parkin – Parkinson’s Disease Related 1 (PDR-1). We performed protein interactor screen using Co-immunoprecipitation followed by mass spectrometry analysis to identify putative interacting partners of PDR-1. Ribonucleic acid interference (RNAi) screen and an unbiased mutagenesis screen were used to identify genes regulating PDR-1 abundance. Confocal microscopy was used for the identification of the subcellular localization of PDR-1 and alpha-Synuclein processing.

**Results: **We show that the *mCherry::pdr-1* transgene rescues the mitochondrial phenotype of *pdr-1* mutants and that the expressed PDR-1 reporter is localized in the cytosol with enriched compartmentalization in the autophagy-lysosomal system. We determined that the transgenic overexpression of the PDR-1 reporter, due to inactivated small interfering RNA (siRNA) generation pathway, disrupts autophagy-lysosomal dynamics. From the RNAi screen of putative PDR-1 interactors we found that the inactivated Adenine Nucleotide Translocator *ant-1.1/hANT*, or hybrid ubiquitin genes *ubq-2/h* *UBA52* *and* *ubl-1/h* *RPS27A* encoding a single copy of ubiquitin fused to the ribosomal proteins L40 and S27a, respectively, induced PDR-1 abundance and affected lysosomal dynamics. In addition, we demonstrate that the abundant PDR-1 plays a role in alpha-Synuclein processing.

**Conclusions:** These data show that the abundant *C. elegans* Parkin ortholog affects the autophagy-lysosomal system together with alpha-Synuclein processing which can help in understanding the pathology in Parkin-related diseases.

## Plain language summary

Parkin promotes activity of the cell cleaning machinery by targeting specific damaged proteins and organelles for their degradation, which allows cells, tissues and whole organisms to cope with stress and aging. Individuals carrying genetic mutations in the gene encoding Parkin develop Parkinson’s disease at a young age likely due to disrupted processing of the neuronal protein alpha-Synuclein. This phenomenon is evolutionarily conserved because animal models, such as mice, fish or flies, exhibit progressive alpha-Synuclein pathology upon Parkin inactivation. Here, we generated a worm model with fluorescently labeled Parkin, which, when accumulated in the cells, worsened that pathology associated with Parkinson’s disease. This study shows that mutations that cause excessive Parkin accumulation in the roundworm *C. elegans* resemble some characteristics of aging-related diseases and could therefore explain the cellular pathology of some Parkin-related diseases, such as Parkinson's disease or cancer.

## Introduction

Various cellular responses to stress conditions that can promote either their survival, or death through apoptosis, have evolved to maintain the health of the whole organism. Parkin is an evolutionarily conserved E3-ubiquitin ligase with a broad role in cell stress by targeting various proteins for degradation through ubiquitin-proteasomal system ^1^. Several loss-of-function mutations in the human *Parkin RING-in-between-RING (RBR) E3 Ubiquitin Protein Ligase (PRKN)* gene, which encodes Parkin, have been associated with the autosomal recessive juvenile form of Parkinson’s disease (PD), a devastating disease due to loss of dopaminergic neurons in the midbrain Substantia Nigra ^2^. Since disease-causing mutations are spread across all exons, and may thus affect several aspects of Parkin biology, including autoinhibition, translocation, interaction or ligase activity and its regulation, it raises the possibility of distinct pathogenetic mechanisms induced by various Parkin mutants that occur in different domains of the protein ^3^. Indeed, some mutations, such as R275W, appear to have gain-of-function neurotoxic properties with dominant inheritance highlighting the fact that sometimes heterozygous carriers may develop PD ^4^.

Parkin is expressed in the cytosol and possesses several regulatory and functional domains. The N-terminal part of the protein encodes an autoinhibitory ubiquitin-like domain (Ubl), that structurally resembles ubiquitin (Ub), which is followed by four zinc-coordinating Really Interesting New Gene (RING)-like domains (RING0, RING1, IBR and RING2) that mediate ubiquitin ligase activity ^5^. How Parkin is regulated for selective protein targeting is not well understood. Parkin is autoinhibited under basal conditions and requires activation that has been described for its role in mitophagy (autophagy of the damaged mitochondria) ^6^. Phosphatase And Tensin Homolog Induced Putative Kinase 1 (PINK1), a sensor of the depolarized mitochondria, is a direct activator of Parkin ^3^. Insufficiently imported PINK1 into mitochondria phosphorylates both the Ubl domain of Parkin as well as Ubiquitin at the equivalent residue Serine 65 ^7^. Both phosphorylation events trigger a binding switch between the Ubl domain and Ub that releases autoinhibition through opening of the E2-binding site in RING1 and the catalytic site in RING2 ^8^. Activated Parkin is then recruited onto the outer mitochondrial membrane where it mediates ubiquitination of proteins to trigger mitophagy ^9^.

Functional *C. elegans* Parkin ortholog, that structurally resembles all Parkin domains, including regulatory sites targeted by PINK1 and phospho-Ubiquitin, is encoded by the *pdr-1* gene ^10^ and regulates accumulation of mitochondrial DNA mutations, mitochondrial fusions as well as the mitochondrial untranslated protein response ^11– 13^. Next to PDR-1’s conserved role in mitochondrial quality control, it also targets the small guanine triphosphatases (GTPase) CED-10 (Cell Death Abnormality) to regulate cytoskeletal rearrangements preventing apoptotic cell engulfment ^14^.

The activation of the Parkin towards non-mitochondrial targets, such as synaptic endophilin and synaptojanin ^15, 16^, endo-lysosomal GTPase Rab7 (Rat Sarcoma Virus-associated binding) ^17^ or alpha-Synuclein ^18^ are unclear. Several phosphorylation sites, S-nitrosylation and S-sulfhydration have been detected though the effect of these modification needs further studies ^19, 20^. Here, we used the genetics of *C. elegans* to seek regulators of *C. elegans* Parkin ortholog PDR-1. We generated a new functional mCherry reporter of PDR-1 and identified regulators of its abundance. We show that abundant PDR-1 reporter impacts autophagy-lysosomal dynamics and alters processing of exogenously expressed human alpha-Synuclein in *C. elegans*.

## Methods

### *C. elegans* strains

Animals were maintained under standard procedure with nematode growth media (NGM) plates unless otherwise stated. The *C. elegans* construct for *dmaIs48* transgene was generated by Invitrogen Gateway recombination cloning technology. Specifically, we cloned the entire *pdr-1* gene, which was amplified by direct polymerase chain reaction (PCR) using 5’-agGGAAGTGGCTCGAGTATGTCTGATGAAATCTCTATA-3’ and 5’-GGCCGATGCGGAGCTCTTAATTAAACCAATGGTCCCATT-3’ primers, into *pDEST-mCherry* vector in frame to *mCherry* reporter gene with *unc-54 3'-untranslated region (3'UTR)*. The *pDEST-mCherry::pdr-1-unc-54 3'-UTR* vector was subsequently recombined with pENTRY vector carrying *rpl-28* promoter sequence. Transgenic strains were generated by germline transformation using microinjection technique. Transgenic construct was injected at 50 ng/μl into the Bristol strain N2 (20 individual nematodes were used) and stable extrachromosomal lines of mCherry positive animals were established. Extrachromosomal array was subsequently integrated by ultraviolet (UV) irradiation (5 individual nematodes were used) and the strain carrying *dmaIs48* was 2x outcrossed.

The strains used were as follow: wild isolate N2, *xmSi(mai-2p::mai-2::GFP::mai-2 3’-UTR), xmSi;pdr-1(gk448), xmSi;drp-1(tm1108), xmSi;eat-3(ad426), xmSi;dmaIs48(rpl-28p::mCherry::pdr-1);pdr-1(gk448), dmaIs48;xmSi, dmaIs48;xmSi; rde-1(dma341), dmaIs48;adIs2122(lgg-1p::GFP::lgg-1), dmaIs48;adIs2122; rde-1(dma341), dmaIs58(rpl-28p::lmp-1::GFP), dmaIs48;dmaIs58, dmaIs48;dmaIs58;rde-1(dma341), uonEx1(alpha-Synuclein::YFP), dmaIs48;uonEx1(alpha-Synuclein::YFP).*

### Imaging and image analysis

Animals were mounted onto a 2% agarose pad containing 10 mM sodium azide and imaged with an EVOS FL auto digital microscope for epifluorescence imaging or a confocal LeicaSP8-X confocal laser scanning microscope within 2–5 minutes. For anoxia stress assay, animals were placed into hypoxia incubator chamber ( StemCell, 27310) with constant nitrogen flow delivery to achieve nearly 0% oxygen for 6 or 24 hours prior the imaging ^21^. For Carbonyl cyanide p-trifluoro-methoxyphenyl hydrazone (FCCP) assay, animals were exposed to 10uM FCCP 1 hour before imaging. ImageJ (Fiji) was used for the quantification of the fluorescent signal and image processing. Identical setting and conditions were used to compare experimental groups with controls. For the quantification of alpha-Synuclein inclusions, we measured size of the fluorescent foci in the body wall muscle cells from the confocal images using Fiji. At least three images representing each condition from three independent biological replicates were analyzed.

### Forward genetic screen

Forward genetic screen for altered *pdr-1* reporter mutants after ethyl methanesulfonate (EMS)-induced random mutagenesis was performed as described previously ^22, 23^. To screen for mutations that alter expression pattern of the mCherry::PDR-1 reporter, we mutagenized 120 L4 animals carrying the *dmaIs48; xmSi[mai-2::GFP]* with 50 mM EMS in M9 for four hours at 20°C with constant rotation. Worms were subsequently washed in M9 and placed on new NGM plates. We observed the F2 progeny for phenotype using a fluorescence dissecting microscope ( Nikon SMZ800N). Animals with constitutively bright mCherry fluorescence were isolated and subsequently sequenced by whole-genome sequencing to obtain lists of candidate genes. Whole genome sequencing was carried out at the UCSF Genomics Facility. DNA sequences from each mutant were analyzed using the CloudMap pipeline Unmapped Mutant Workflow ^24^ available on the open-source Galaxy web-based platform ^25^. Ribonucleic acid interference (RNAi) directed against genes with putative causal mutations was performed to confirm phenocopying.

### RNA interference

Feeding RNAi was performed as previously described ^26^. Five gravid animals carrying *dmaIs48* transgene were placed on NGM media containing ampicillin 25 μg/ml and 1mM Isopropyl β-d-1-thiogalactopyranoside (IPTG) and seeded with bacteria producing the desired double stranded RNA (dsRNA). Progeny were subsequently grown at 23°C and screened for the phenotype in young adult stage. To induce mild RNAi against *ant-1.1*, *ubq-2* and *ubl-1*, that induced larval arrest in the screened progeny under standard procedure, L1 and L2 animals were placed on RNAi plates and screened for the phenotype in young adult stage. Visual examination of the animals was done on fluorescent stereoscope ( Nikon SMZ800N). The bacterial clones were obtained from *C. elegans* RNAi collection - Ahringer ( Source Bioscience, 3318).

### Western blot analysis

Animals were lysed in the Laemmli sample buffer ( Bio-Rad, 1610747) supplemented with the reducing agent *β*-mercaptoethanol, followed by boiling the samples for 10 min. The worm lysates were separated on 4–15% sodium dodecyl sulfate-polyacrylamide gel electrophoresis (SDS PAGE) ( Bio-Rad, 4561086). The proteins were transferred to a nitrocellulose membrane ( Bio-Rad, 1620167) and subsequently detected by anti-mCherry rat monoclonal antibody ( ThermoFisher Scientific, M11217 1:1000), anti-Synuclein Mouse monoclonal antibody ( Abnova, MAB5383 1:2000) and re-incubated with anti-histone H3 rabbit polyclonal antibody ( Abcam, ab1791 1:2000) as a loading control.

### Co-Immunoprecipitation (Co-IP) Analyses

Whole-animal extracts ( *dmaIs48; xmSi(mai-2::GFP); rde-1(dma341)*) were prepared by sonication in M9 buffer (22 mM KH2PO4, 42 mM Na2HPO4, 86 mM NaCl) with protease/phosphatase inhibitor mixture ( Sigma-Aldrich, 11836153001 1x). Centrifugation-cleared animal extracts were precleaned by control magnetic beads ( bmab-20, ChromoTek) for 30 mins at 4˚C, and followed by immunoprecipitation with RFP-trap magnetic beads ( ChromoTek, rtma-10) at room temperature for one hour. The RFP-trap beads were subsequently washed five times with M9 buffer. Co-immunoprecipitated proteins were eluted by heating at 70 °C in Laemmli buffer ( Bio-Rad, 1610747) for 15 min. Eluates were then subjected to SDS-PAGE and stained with Coomassie Blue to visualize the bands for subsequent in-gel digestion.

### Protein identification using liquid chromatography coupled with tandem mass spectrometry (LC-MS/MS)

LC-MS/MS analysis following an in-gel digestion was performed by International Research Resource Center in Biomolecular Mass Spectrometry and Proteomics at University of California San Francisco (UCSF). The in-gel digestion was carried out using their standard sample preparation procedure, available here. The proteins in each gel band were reduced, alkylated, and finally digested overnight with 100 ng of Trypsin ( Promega, v511c). The resultant peptide mixture was desalted with µC18-ZipTips ( Millipore, ZTC18M960), speed vacuum dried, suspended in 0.1% formic acid, and analyzed on a Velos Pro Elite Orbitrap Mass Spectrometer. The mass spectrometric data was obtained in a data-dependent acquisition mode. The data was then converted into peak lists with PAVA ^27^, a software developed by International Research Resource Center in Biomolecular Mass Spectrometry and Proteomics. The raw data can be also processed by software package MaxQuant. Using ProteinProspector search engine (v5.20.1), the peak lists were searched against the SwissProt *C. elegans* database and the proteins detected by the LC-MS/MS were subsequently identified.

## Results

### *C. elegans* Parkin reporter colocalizes with autophagy-lysosomal compartments

**Figure 1.**
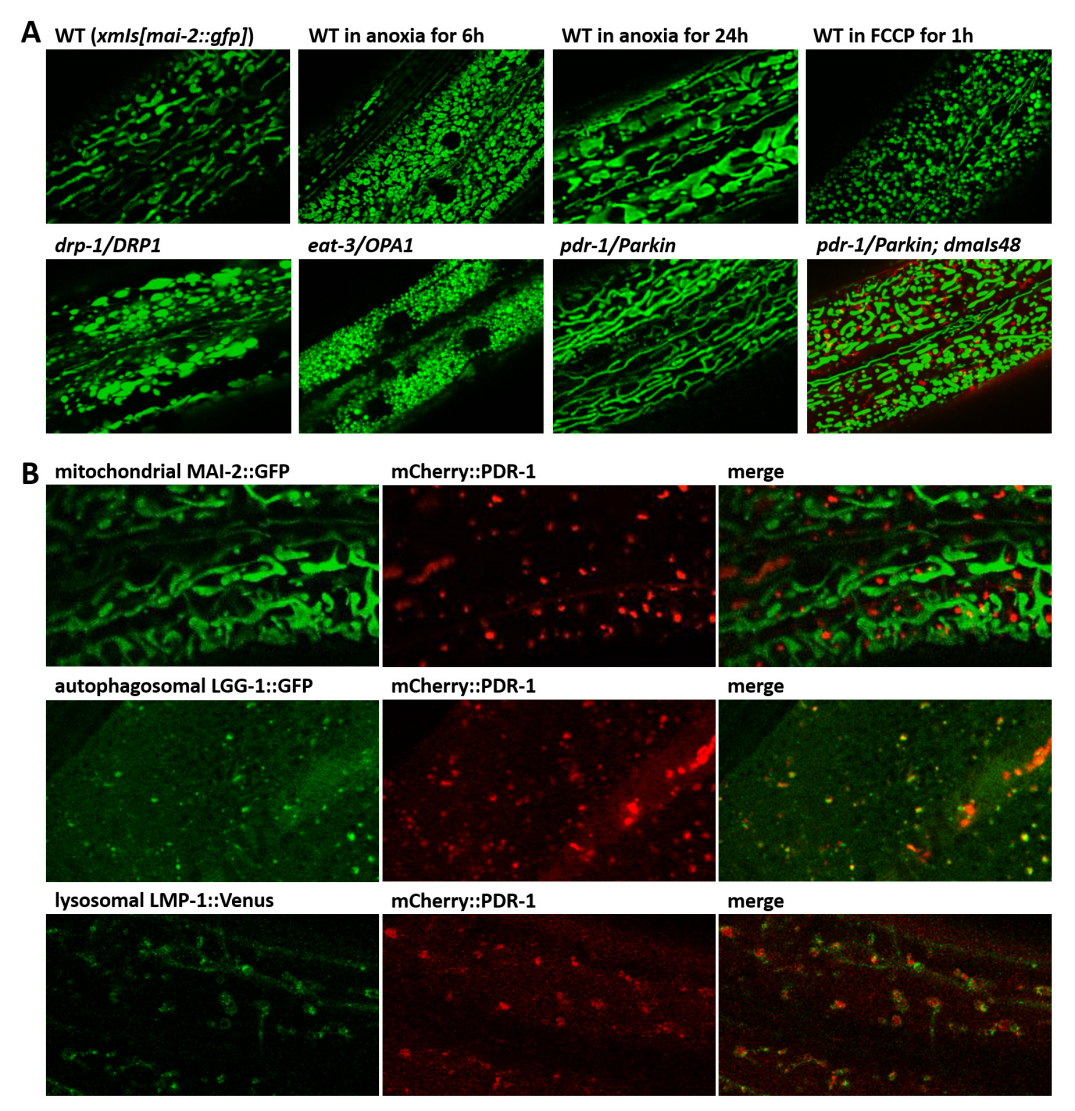
The mCherry reporter of *C. elegans* Parkin (PDR-1) is enriched in the autophagy-lysosomal system. **A.** Exemplar green fluorescent protein (GFP) fluorescence confocal images showing mitochondrial morphology in the hypoderm of *xmSi(mai-2::GFP)* animals under several environmental and genetic stresses. The PDR-1 reporter expressed from *dmaIs48* transgene restores mitochondrial pattern in *pdr-1* mutants. **B.** Confocal GFP and mCherry fluorescence images showing expression pattern of the mCherry::PDR-1 together with mitochondrial reporter *xmSi(mai-2::GFP)*, autophagosomal reporter *adIs2122(lgg-1::GFP)* and lysosomal reporter *dmaIs58(lmp-1::Venus)* in the hypoderm. The PDR-1 reporter colocalizes with the autophagosomal and lysosomal reporters.

To study regulatory pathways of the Parkin we first investigated the mitochondrial morphology in *pdr-1* mutant animals using mitochondrial reporter *xmSi* encoding Mitochondrial ATPase Inhibitor fused to Green Fluorescent Protein (MAI-2::GFP) ^28^. We focused on the hypodermal tissue in adult animals, the largest multinucleated syncytial cell in *C. elegans*. We found that *pdr-1* deficiency leads to elongation of the mitochondrial pattern as seen previously using different mitochondrial reporters ^12, 13^. We compared such morphology with other known mediators of the mitochondrial dynamics. First, we examined the mitochondrial morphology in the genetic mutants of *drp-1* (Dynamin-Related Protein) encoding the ortholog of DRP1 whose activity is required for the mitofission and *eat-3* (Eating: abnormal pharyngeal pumping) encoding ortholog of Optic atrophy 1 (OPA1) whose activity is required for mitofusion. As expected, while *drp-1* mutants exhibited large disorganized mitochondrial stains, the *eat-3* mutants exhibited fragmented mitochondrial pattern. Next, we investigated mitochondrial morphology in animals subjected to anoxic conditions and mitochondrial oxidative phosphorylation uncoupler FCCP (Carbonyl cyanide p-trifluoro-methoxyphenyl hydrazone). Interestingly, animals exposed to six hours of anoxia (near to 0% oxygen) exhibited donut-like shapes while animals exposed to 24 hours of anoxia exhibited hypermitofusion characterized by the enlarged mitochondria of various shapes. On the other hand, the FCCP triggered fragmented round shape morphology, that differs from the mitochondrial fragmentation in *eat-3* mutants, highlighting mitochondrial shape under disrupted membrane potential. These data show that induced mitochondrial elongations due to loss of *pdr-1*/Parkin differ from the anoxia-induced mitochondrial fusions as well as from disrupted fragmentation in *drp-1* mutants ( Figure 1A).

We used the *pdr-1* phenotype to validate the newly generated mCherry reporter of PDR-1. We cloned the entire *pdr-1* gene in frame behind the mCherry reporter under ubiquitous promoter *rpl-28*. Using confocal microscopy we found that the newly generated transgenic strain carrying *dmaIs48(rpl-28p::mCherry::pdr-1)* exhibits bright fluorescent foci of mCherry::PDR-1 that do not colocalize with GFP reporter for mitochondria MAI-2::GFP, though still restored mitochondrial morphology in *pdr-1* mutants ( Figure 1A, 1B). Since mCherry::PDR-1 appears to be a functional Parkin reporter, we investigated whether the mCherry::PDR-1 reporter recruits onto mitochondria upon exposure to FCCP by following the PDR-1 subcellular localization for 30 minutes after the exposure to FCCP. Although the FCCP altered the mitochondrial pattern that formed round shape morphology, we did not observe recruitment of the mCherry::PDR-1 onto mitochondria despite its dynamic expression pattern in both standard and stressed conditions; see Figure 1A, and Supplementary Video 1 and 2 in *Extended data* ^29^. To determine the subcellular localization of the mCherry::PDR-1 foci we crossed the *dmaIs48* transgene with the GFP reporter for autophagosome *adIs2122(lgg-1p::GFP::lgg-1)* ^30^ and GFP(Venus) reporter for lysosome *dmaIs58(rpl-28p::lmp-1::Venus)* ^31^. The confocal microscopy showed that the mCherry foci colocalize with both (LC3, GABARAP and GATE-16 family) and LMP-1 (LAMP (lysosome-associated membrane protein) homolog) reporters indicating that mCherry::PDR-1 is targeted to autophagosome-lysosome compartments ( Figure 1B). The full data associated with the results are available in *Underlying data* ^29^.

### Endogenous RNAi pathway regulates expression of mCherry::PDR-1

**Figure 2 .**
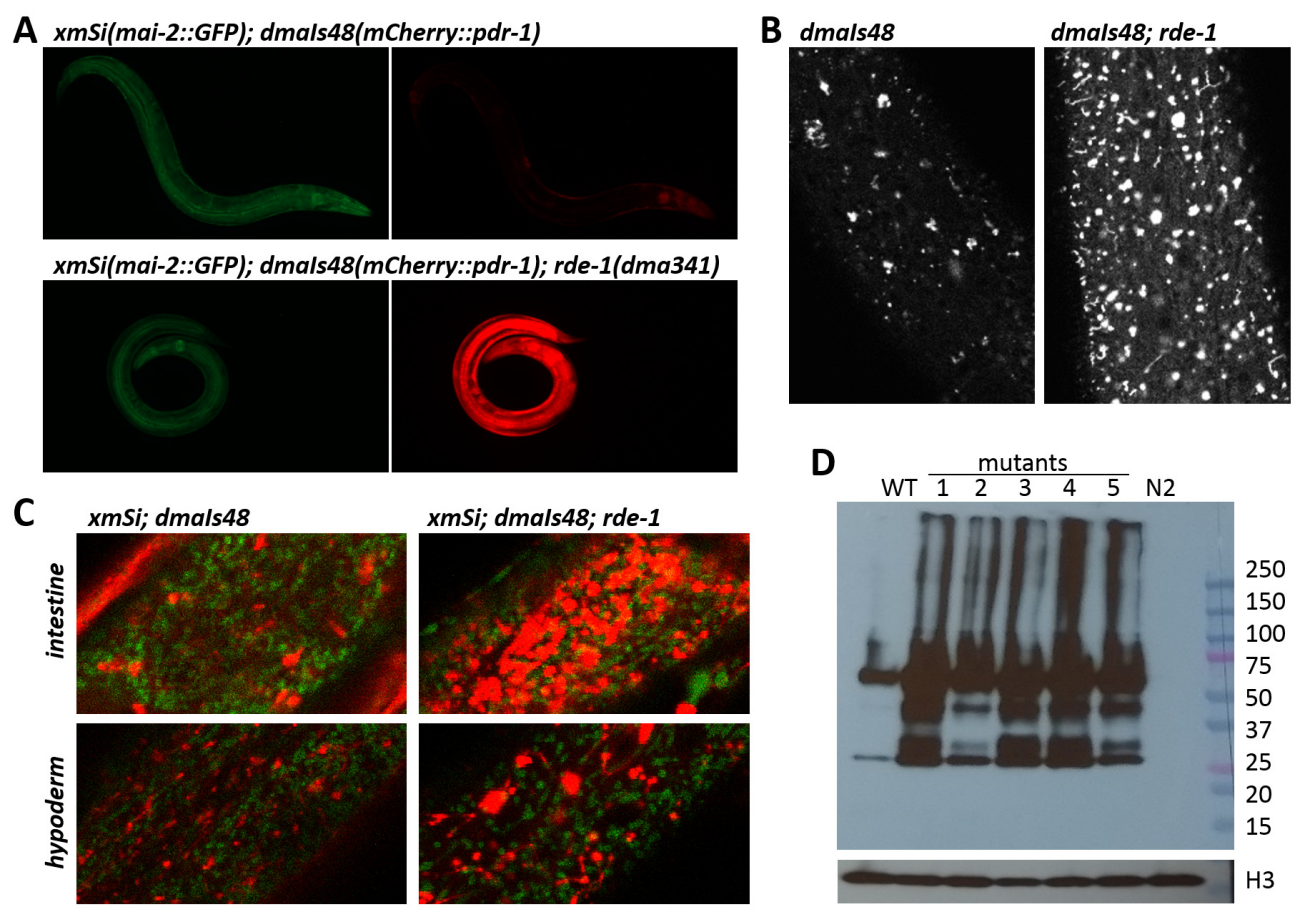
The abundant *C. elegans* Parkin (PDR-1) reporter in ethyl methanesulfonate (EMS)-derived mutants. **A.** Exemplar green fluorescent protein (GFP) (left) and mCherry (right) fluorescence images showing mCherry::PDR-1 and MAI-2::GFP expression in WT and *rde-1(dma341)* mutant. Only mCherry::PDR-1 expression is increased in *rde-1* mutants. **B.** Fluorescence confocal images of the hypoderm showing the expression pattern of mCherry::PDR-1 in WT and *rde-1(dma341)* mutant. **C.** Confocal view of merged MAI-2::GFP and mCherry::PDR-1 fluorescence in intestine (upper) and hypoderm (lower) of WT and *rde-1(dma341)* mutant. **D.** The mCherry::PDR-1 protein levels determined by sodium dodecyl sulfate-polyacrylamide gel electrophoresis (SDS PAGE) followed by western blot analysis using anti-mCherry Ab and anti-histone H3 Ab. *C. elegans* protein lysates from various genetic backgrounds were analyzed. 50 µg protein samples were loaded per lane. WT represents parental strain carrying *dmaIs48(mCherry::pdr-1)*. Analyzed mutants with *dmaIs48* are as follows: 1. *rde-1(dma325)*, 2. *ego-1(dma327)*, 3. *rrf-1(dma339)*, 4. *rde-1(dma341)*, 5. *mut-2(dma340).* N2 represents wild isolate N2.

Because of the striking mCherry::PDR-1 expression pattern in *dmaIs48* animals we aimed to identify novel regulators of the PDR-1 subcellular expression by unbiased approaches. We performed a forward genetic screen using EMS mutagenesis to isolate mutants with an altered expression pattern. From a screen of approximately 100,000 haploid genomes of the *dmaIs48; xmSi(mai-2::GFP)* strain we have isolated 10 independent mutants with 100% penetrance of markedly increased red fluorescent signal while the GFP signal was not altered ( Table 1, Figure 2A). Confocal microscopy showed that the mutants exhibit increased fluorescent signal in the cytosol as well as increased number and size of the foci ( Figure 2B) and revealed accumulated mCherry::PDR-1 reporter across the tissues with a strong signal in intestine and hypoderm ( Figure 2C). Interestingly, the fluorescent foci of the accumulated mCherry::PDR-1 in the EMS-derived mutants lost the dynamic movements in the hypodermal tissue; see Supplementary Video 3 in *Extended data* ^29^. Western-Blot analysis using an anti-mCherry antibody showed increased PDR-1 reporter levels in the mutants accompanied with partial processing of the PDR-1 reporter, which is indicated by detection of the shorter variants, compared to parental strain ( Figure 2D). Whole genome sequencing of the isolated mutants revealed that 4 mutants carry the protein-changing mutations in the *rrf-1* gene. The *rrf-1* encodes RNA-directed RNA polymerase and is a component of the WAGO 22G RNAs RNAi pathway that generate siRNA for transcriptional silencing of exogenous RNAs ^32^. Notably, all the remaining isolated mutants carry at least one protein-changing mutation in the genes of the RNAi pathway, such as *rde-1*, *rde-10*, *ego-1*, *mut-2* and *mut-16* ( Table 1). To verify causality of these mutations we employed RNAi against *rrf-1*, *rde-1* and *mut-16* using parental strain and, indeed, we observed that all three RNAi conditions increased reporter levels and thus phenocopied isolated mutants. These data show that increased levels of the mCherry::PDR-1 in the isolated EMS-derived mutants are due to loss of transgene silencing activity at the post-transcriptional level. The full data associated with the results are available in *Underlying data* ^29^.

**Table 1.** Ethyl methanesulfonate (EMS)-derived mutants with abundant Parkin reporter mCherry::PDR-1.

mutation	chr.	penetrance	Gene	Protein change	Annotation
*dma334*	I	100%	*rde-10*	splicing	RNA interference defective protein
*dma330*	I	100%	*mut-2*	W164STOP	Mutator
*dma337*	I	100%	*rrf-1*	Q52STOP	RNA-dependent RNA polymerase
*dma338*	I	100%	*rrf-1*	A944T	RNA-dependent RNA polymerase
*dma339*	I	100%	*rrf-1*	G648R	RNA-dependent RNA polymerase
*dma330*	I	100%	*rrf-1*	R1256STOP	RNA-dependent RNA polymerase
*dma327*	I	100%	*ego-1*	D1305N	RNA-dependent RNA polymerase
*dma333*	I	100%	*mut-16*	Q808STOP	Mutator
*dma341*	V	100%	*rde-1*	P963L	Component of RNA-induced silencing complex
*dma325*	V	100%	*rde-1*	splicing	Component of RNA-induced silencing complex

### Hybrid ubiquitins and mitochondrial ANT and ATPase regulate mCherry::PDR-1 levels

**Figure 3.**
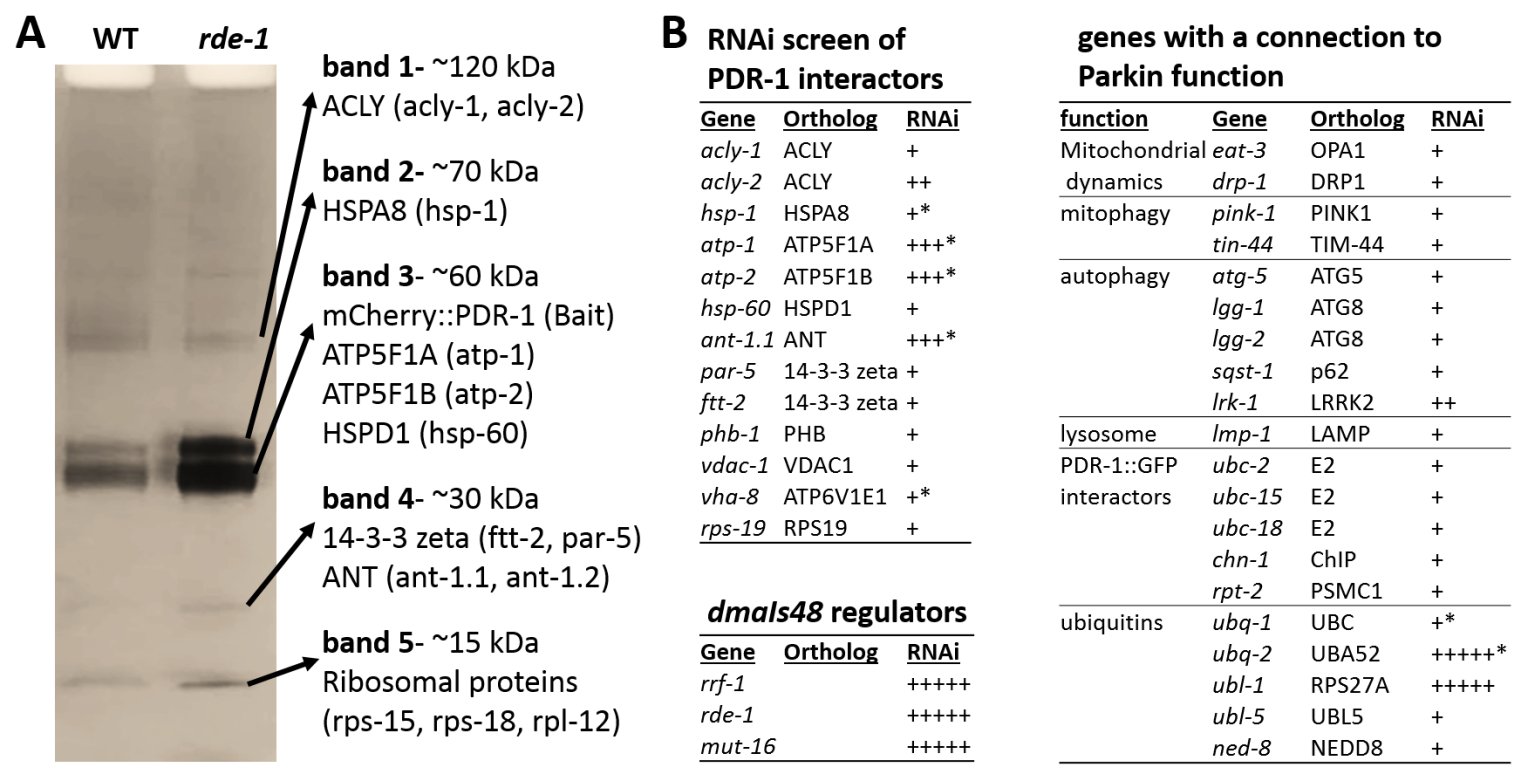
Protein interactor and RNAi screen identify regulators of *C. elegans* Parkin (PDR-1) abundance. **A.** Sodium dodecyl sulfate-polyacrylamide gel electrophoresis (SDS PAGE) followed by Coomassie staining of mCherry::PDR-1 pull down. Red fluorescent protein (RFP)-trap was used for Co-immunoprecipitation of protein extracts from *dmaIs48(mCherry::pdr-1)* and *dmaIs48;rde-1(dma341)* animals. Mass spectrometry (MS) analysis of individual bands was used to identify putative interactors. Proteins with the highest peptide coverage are indicated. **B.** Ribonucleic acid interference (RNAi) screen of indicated genes for abundant mCherry::PDR-1.The “plus” symbol indicates mCherry fluorescence intensity. The “star” symbol indicates larval arrest phenotype.

Although unbiased mutagenesis revealed regulators of the transgenic mCherry::PDR-1 expression, novel alleles regulating PDR-1 function have not been isolated. Therefore, we employed Co-immunoprecipitation (CoIP) using RFP-trap to identify putative interacting proteins with the mCherry::PDR-1 reporter. We used crude extract of both parental strain *dmaIs48* as well as isolated mutant *dmaIs48; rde-1(dma341)* as the baits. One-dimensional sodium dodecyl sulfate-polyacrylamide gel electrophoresis (SDS PAGE) revealed five bands of protein pull down that did not differ in size between parental and mutant samples ( Figure 3A). We analyzed these bands by gel digest followed by liquid chromatography-tandem mass spectrometry (GeLC-MS/MS) analysis and identified putative interactors with the highest score for adenosine triphosphate (ATP)-Citrate Lyase ACLY-1/hACLY, Heat Shock 70kDa HSP-1/hHSPA1 and hHSPA8, 14-3-3-like proteins PAR-5/hYWHAZ and FTT-2/hYWHAZ and several Ribosomal Proteins. Notably, subunits of the Vacuolar ATPase (VHA-8, VHA-12, VHA-13), and several mitochondrial proteins, such as mitochondrial Adenine Nucleotide Translocator ANT-1.1/hANT, ATP synthase subunits ATP-1/hATP5F1A and ATP-2 hATP5F1B, Voltage Dependent Anion Channel VDAC-1/hVDAC1, or Heat Shock Protein HSP-60/hHSPD1, have been also identified; see Supplementary Table 1 in *Extended data* ^29^.

Next, we performed reverse genetic screen by RNAi against putative PDR-1 regulators. First, we have systematically screened the genes encoding the identified PDR-1 interactors for altered expression pattern of the mCherry::PDR-1 reporter. We found that mCherry::PDR-1 abundance was enhanced by RNAi against mitochondrial *ant-1.1*, *atp-1* and *atp-2* accompanying with developmental arrest at early stages of larva development. Because ANT has been recently identified driver of mitophagy trough Parkin activity ^33^, we hypothesized that impaired mitophagy may lead to Parkin accumulation. However, the RNAi against *pink-1/*hPINK1 and *tin-44/*hTIM44 (Transport to Inner Mitochondrial Membrane (yeast TIM)), the components of the ANT/Parkin-mediated mitophagy did not increase the expression of mCherry::PDR-1. We have employed RNAi against other genes with a connection to Parkin function, including previously identified PDR-1::GFP interactors ^10^ or genes encoding ubiquitin, and found that inactivation of hybrid ubiquitin genes *ubl-1* (Ubiquitin-like) and *ubq-2* (ubiquitin) increased the abundance of mCherry::PDR-1, accompanying with developmental arrest at early stages of larva development. These data suggest that accumulation of the PDR-1 reporter is not related to impaired initiation of mitophagy but rather due to impaired mitochondrial function, ubiquitination or proteasomal degradation. The full data associated with the results are available in *Underlying data* ^29^.

### Accumulated mCherry::PDR-1 disrupts lysosomal dynamics

**Figure 4.**
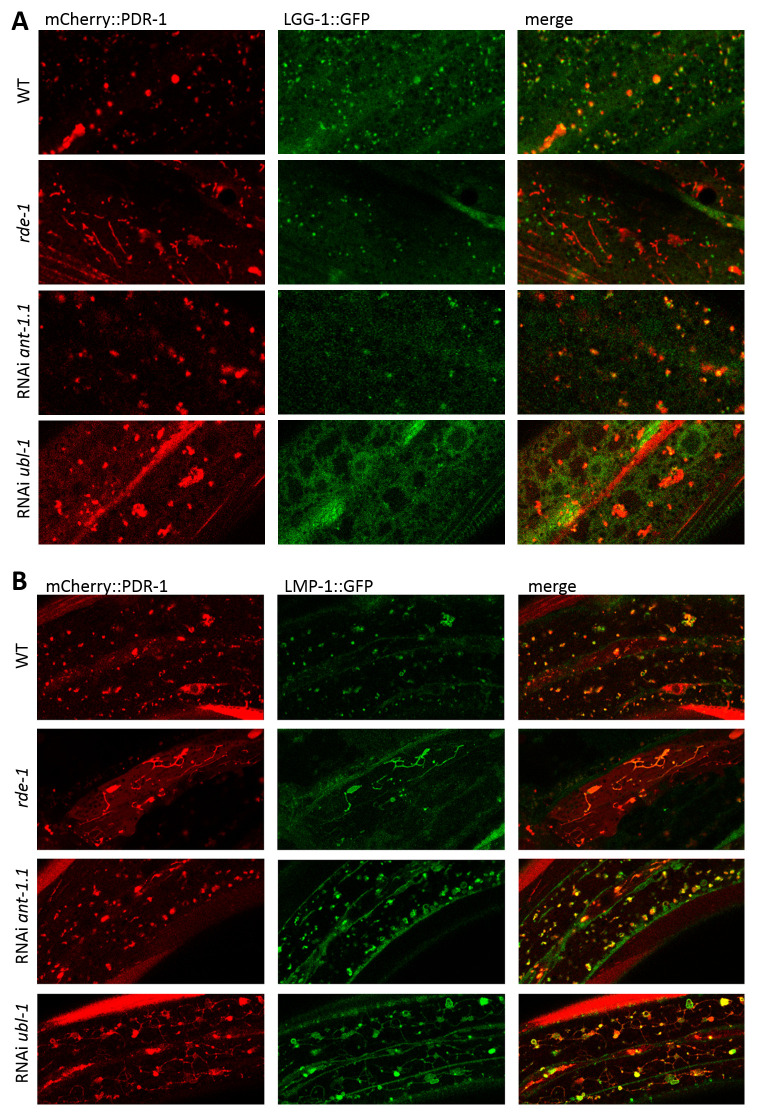
Abundant *C. elegans* Parkin (PDR-1) reporter alters lysosomal morphology. **A.** Enlarged view of confocal green fluorescent protein (GFP) and mCherry fluorescence images showing expression pattern of the autophagosomal reporter *adIs2122(lgg-1::GFP)* and PDR-1 reporter *dmaIs48(mCherry::pdr-1)* in the hypoderm of indicated mutants/knockdowns. **B.** Enlarged view of confocal GFP and mCherry fluorescence images showing lysosomal reporter *dmaIs58(lmp-1::Venus)* and PDR-1 reporter *dmaIs48(mCherry::pdr-1)* in the hypoderm of indicated mutants/knockdowns.

**Figure 5.**
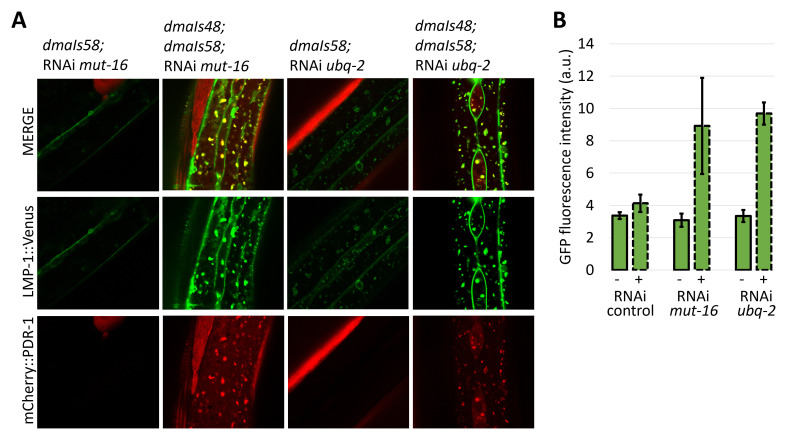
Abundant *C. elegans* Parkin (PDR-1) reporter increases expression of lysosomal reporter LMP-1. A. Confocal green fluorescent protein (GFP) and mCherry fluorescence images showing lysosomal reporter *dmaIs58(lmp-1::Venus)* in the absence and presence of the PDR-1 reporter *dmaIs48(mCherry::pdr-1)* upon various ribonucleic acid interference (RNAi) assay. **B.** Quantification of the GFP fluorescence intensity detected in the widefield image of animals carrying *dmaIs58(lmp-1::Venus)* in the absence (-) and presence (+) of the *dmaIs48(mCherry::pdr-1)* upon various RNAi assay. 3 animals from independent biological replicates were analysed.

Since increased expression of the PDR-1 reporter in the *rde-1* mutants lost its dynamic pattern in the hypoderm we asked whether its subcellular localization differ compared to the parental strain. We crossed the *mCherry::pdr-1* reporter with autophagy *lgg-1::GFP* and hypodermal *lmp-1::Venus* reporters and found that accumulated PDR-1:mCherry in the *rde-1* mutants does not colocalize with LGG-1:GFP reporter, though the *ant-1.1* and *ubl-1* knockdowns upon mild RNAi still showed partial colocalization ( Figure 4A). Interestingly, all conditions, including *rde-1(dma341),* RNAi *ant-1.1* and RNAi *ubl-1*, induced expression of both mCherry::PDR-1 and LMP-1::Venus reporters when expressed together, and colocalized in the lysosomal-like structures. Notably, the dynamics of the PDR-1 reporter in *ubl-1* knockdowns was interrupted (Supplementary Video 4, *Extended data* ^29^) compared to control and the LMP-1::Venus expression was increased at the plasma membrane where mCherry::PDR-1 has not been detected ( Figure 4A). Because the *lmp-1::Venus* transgene also uses the same ubiquitous promotor *rpl-28* as *mCherry::PDR-1*, we asked whether accumulated LMP-1::Venus in animals with accumulated mCherry::PDR-1 is dependent on the co-expressed *mCherry::pdr-1*. We employed RNAi against *mut-16* and *ubq-2*, the most striking RNAi conditions that induce mCherry::PDR-1 accumulation, in the animals carrying only the *lmp-1::Venus* transgene. We found that expression of LMP-1::Venus was not induced and retained its dynamics in respective knockdowns indicating that the accumulation of the LMP-1::Venus is dependent on the co-expression of the mCherry::PDR-1 ( Figure 5A and 5B). These data indicate that lysosomal dynamics are disrupted due to the abundant mCherry::PDR-1.The full data associated with the results are available in *Underlying data* ^29^.

### The mCherry::PDR-1 alters formation of alpha-Synuclein inclusions

**Figure 6.**
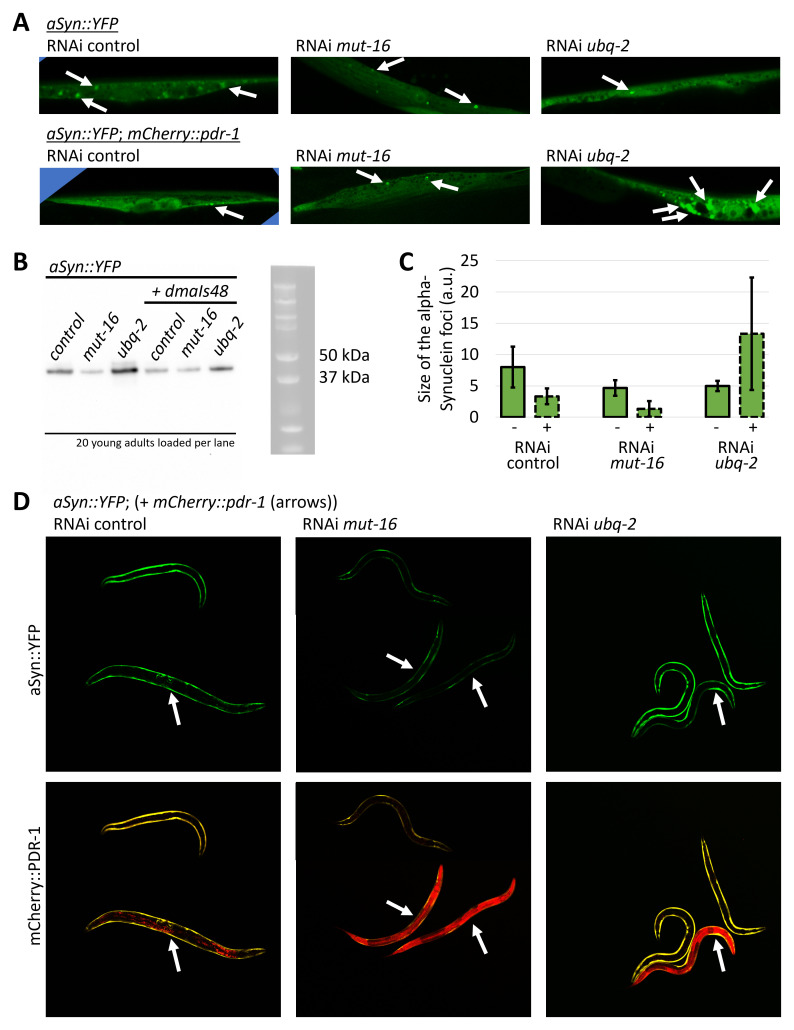
The *C. elegans* Parkin (PDR-1) alters alpha-Synuclein processing. **A.** Representative confocal green fluorescent protein (GFP) fluorescence images of the body wall muscle cells in animals carrying uonEx1(alpha-Synuclein::YFP) in the absence and presence of the *dmaIs48(mCherry::pdr-1)* upon various ribonucleic acid interference (RNAi) assay. **B** The alpha-Synuclein::YFP protein levels as determined by sodium dodecyl sulfate-polyacrylamide gel electrophoresis (SDS PAGE) followed by western blot using anti-alpha-Synuclein Ab (Abnova MAB5383). Twenty young adult animals collected from three biological replicates upon various RNAi were loaded per lane. **C.** Size quantification of detected fluorescent foci in body wall muscle cells expressing alpha-Synuclein::YFP in the absence (-) and presence (+) of the *dmaIs48(mCherry::pdr-1)* upon various RNAi assay. 3 body wall muscle cells from independent biological replicates were analysed. **D.** Exemplar GFP and mCherry fluorescence images of animals carrying *uonEx1(alpha-Synuclein::YFP)* in the absence and presence of the *dmaIs48(mCherry::pdr-1)* upon indicated RNAi assay. Arrows indicate animals carrying *dmaIs48(mCherry::pdr-1)* which exhibit reduced GFP fluorescence associated with expression levels of alpha-Synuclein reporter.

We then questioned whether disrupted autophagy-lysosomal dynamics in animals with accumulated mCherry::PDR-1 in cytosol and lysosomes alters proteostasis in the *C. elegans* model of the Parkinson’s disease. We used a previously well-established GFP-based reporter of alpha-Synuclein aggregation in body wall muscle cells *uonEx1(unc-54p::alpha-Synuclein::YFP)* and with confocal microscopy quantified the size of the YFP fluorescent foci in the presence and absence of the overexpressed mCherry::PDR-1. First, we determined that the size of the foci (inclusions of aggregated signal) was significantly reduced in the young adult animals co-expressing mCherry::PDR-1. In addition, fluorescent microscopy together with western blot analysis revealed that the expression levels of the alpha-Synuclein::YFP were reduced in the presence of the mCherry::PDR-1 ( Figure 6). Next, we asked whether animals with accumulated mCherry::PDR-1, such as *mut-16* and *ubq-2* knockdowns, would further reduce expression of the alpha-Synuclein::YFP. We found that RNAi against *mut-16* reduced expression levels of alpha-Synuclein::YFP in the absence as well as in the presence of the mCherry::PDR-1 accompanied with reduction of the YFP foci. These data suggest that reduction of formed alpha-Synuclein::YFP foci is a result of its reduced expression levels. Interestingly, the RNAi against *ubq-2* increased expression levels of alpha-Synuclein::YFP in the absence of the mCherry::PDR-1, while the *ubq-2* knockdowns with accumulated mCherry::PDR-1 exhibited increased formation of the alpha-Synuclein::YFP foci despite its reduced expression levels ( Figure 6). These data indicate that accumulated mCherry::PDR-1 due to inactivated *ubq-2* alters cellular processing of exogenously expressed alpha-Synuclein. The full data associated with the results are available in *Underlying data* ^29^.

## Discussion

Parkin has an active role during proteotoxic as well as mitochondrial stress. Understanding the regulatory mechanisms distinguishing specific Parkin targets may explain its role in Parkinson’s disease, tumorigenesis, and other Parkin-associated diseases. Here we present a new functional mCherry reporter of *C. elegans* Parkin (PDR-1) that exhibits autophagy-lysosomal targeting and disrupts the dynamic movements of this system upon its intracellular accumulation. We demonstrate that the *mCherry::pdr-1* transgene restored mitochondrial morphology in *pdr-1* mutants, indicating that the mCherry::PDR-1 reporter retains some functionality. On the other hand, we cannot exclude the possibility that mCherry tag at the N-terminal autoinhibitory Ubl domain may alter its regulation, targeting and/or subcellular localization.

Our preliminary protein interactor screen revealed several putative PDR-1 targets, such as VDAC-1 or HSP-1 which are the orthologs of previously identified human Parkin interactors VDAC1 and HSPA8 ^34, 35^. Interestingly, several identified interactors, such as ANT-1.1/hANT and ATP-1/hATP5F1A, are components of the inner mitochondrial membrane, and therefore we suspect that they are targeted by Parkin during the process of mitochondrial turnover, when the outer mitochondrial membrane is ruptured. Congruently, a similar assay with human Parkin identified several mitochondrial proteins whose direct physical interaction is proposed to be taken part during mitophagy after disruption of the mitochondrial membranes ^36^. In fact, Parkin-mediated mitophagy requires both the proteasome system for outer- and autophagy system for inner-mitochondrial membrane rupture ^37^.

From RNAi screen directed against putative PDR-1 interactors we identified hybrid ubiquitin genes *ubq-2* and *ubl-1* or mitochondrial *ant-1.1*, *atp-1 and atp-2* as regulators of the PDR-1 abundance. How exactly these genes maintain mCherry::PDR-1 levels is not clear. Both *ubq-2/hUBA52* and *ubl-1*/ *hRPS27A* encode a single copy of the ubiquitin domain which can be cleaved from the ribosomal subunits L40 and S27a, respectively ^38, 39^. It is therefore possible that UBL-1 and UBQ-2 may serve as the ubiquitin substrates for the ATP-dependent PDR-1 auto-ubiquitination. The western blot analysis of overexpressed PDR-1 reporter indicated its partial processing of the mCherry::PDR-1 which supports this hypothesis. Notably, we also found that inactivation of the *ubq-2* increased levels of the exogenously expressed alpha-Synuclein reporter in body wall muscle cells while the co-expression of the mCherry::pdr-1 reduced alpha-Synuclein levels indicating a role of UBQ-2 along with expressed PDR-1 reporter in the regulation of alpha-Synuclein expression levels. Congruently, beneficial effect of Parkin on alpha-Synuclein toxicity has been demonstrated in midbrain cell cultures ^40^ as well as in a *Drosophila* model of Parkinson’s disease ^41^. Parkin can mediate K63-linked poly-ubiquitination of alpha-Synuclein to trigger its degradation through increased aggregation ^42, 43^. Whether UBQ-2/hUBA52 serves as a ubiquitin substrate for the PDR-1/hParkin ubiquitin ligase activity targeting alpha-Synuclein merits further studies.

Intriguingly, the abundant PDR-1 reporter due to inactivated *ubq-2* altered also alpha-Synuclein processing characterized by enlarged fluorescent foci. Since the RNAi of mitochondrial and ubiquitin genes, which induce abundance of the PDR-1 reporter, results in the developmental arrest we have not been able to investigate whether the enlarged aggregated alpha-Synuclein signal may be beneficial and less toxic than diffused alpha-Synuclein, as observed in other model systems ^44^. Nonetheless, we hypothesize that abundant PDR-1 may sequester intracellular ubiquitin and thus prevent its binding to alternative E3 ubiquitin ligases. Depleted ubiquitin would then result in insufficient ubiquitination of damaged proteins with consequent impairment of the lysosomal dynamics and proteostasis. Interestingly, the abundant PDR-1 along with the affected lysosomal system due to inactivated *mut-16* did not alter alpha-Synuclein processing. We believe that the reduced formation of the aggregated alpha-Synuclein signal in *mut-16* knockdowns is due to reduced expression levels of alpha-Synuclein reporter in these animals.

Interestingly, lysosomal disfunction with altered lysosomal morphology, that resemble lysosomal morphology in *C. elegans* with abundant PDR-1, has been observed also in the Parkin-deficient cell line ^45, 46^ indicating that both inactivated as well as abundant Parkin may affect lysosomal morphology and function. Taken together, since the enlarged inclusions of alpha-Synuclein in our model system resemble Parkinson’s disease pathology, we propose that an affected lysosomal system upon PDR-1 abundance may be one of the possible pathological mechanisms of mutated Parkin.

## Data availability

### Underlying data

Zenodo: *Caenorhabditis elegans* Parkin: regulators of its abundance and role in autophagy-lysosomal dynamics. https://doi.org/10.5281/zenodo.5940065 ^29^.

This project contains the following underlying data:

- List of Underlying Data.docx (contains description of individual underlying data files in each folder).
- Figure 1.zip (The mCherry reporter of C. elegans Parkin (PDR-1) is enriched in the autophagy-lysosomal system).
- Figure 2.zip (The abundant PDR-1 reporter in ethyl methanesulfonate (EMS)-derived mutants).
- Figure 3.zip (Protein interactor and RNAi screen identify regulators of PDR-1 abundance).
- Figure 4.zip (Abundant PDR-1 reporter alters lysosomal morphology).
- Figure 5.zip (Abundant PDR-1 reporter increases expression of lysosomal reporter LMP-1).
- Figure 6.zip (The PDR-1 alters alpha-Synuclein processing).

### Extended data

Zenodo: *Caenorhabditis elegans* Parkin: regulators of its abundance and role in autophagy-lysosomal dynamics. https://doi.org/10.5281/zenodo.5940065 ^29^.

This project contains the following extended data:

- List of Underlying Data.docx (contains description of individual extended data files).
- Extended Data.zip (Supplementary Table 1 and Supplementary Videos 1-5).

Data are available under the terms of the Creative Commons Attribution 4.0 International license (CC-BY 4.0).

## Ethics and consent

In this project we utilize a roundworm *Caenorhabditis elegans* as a genetic model. These microscopic worms are invertebrate and, therefore, they do not fall within the scope of EU Directive 2010/63/EU on the protection of animals used for scientific purposes. No ethical approval was required for this research.
