# Supplementary material for: Fluorescent reporter of  Caenorhabditis elegans Parkin: Regulators of its abundance and role in autophagy-lysosomal dynamics
Source: Open Res Eur. 2023 Sep 15;2:23. Originally published 2022 Feb 18. [Version 2] doi: 10.12688/openreseurope.14235.2 (PMC10556562; doi:10.12688/openreseurope.14235.2)
Supplement: Peer review history

# Reviewer report: Reviewer response for version 2

**DOI:** 10.21956/openreseurope.17936.r35157

**Published:** 2023-10-05

**Authors:** Anat Ben-Zvi

**Affiliations:** 1 Ben-Gurion University of the Negev, Beersheba, Israel

The authors addressed most of my concerns. The new data suggest that the *mCherry::pdr-1* is active but further indicate that the transgene overexpression modulates its function ( *uaDf5* levels in *mCherry::pdr-1* background are 2-fold lower than WT). Thus, the work is clear and technically sound but focuses on the impact of Parkin's overexpression on worms, limiting its applicability to WT PDR-1.

Is the study design appropriate and does the work have academic merit?

Partly

Is the work clearly and accurately presented and does it cite the current literature?

Partly

If applicable, is the statistical analysis and its interpretation appropriate?

No

Are all the source data underlying the results available to ensure full reproducibility?

Yes

Are the conclusions drawn adequately supported by the results?

Partly

Are sufficient details of methods and analysis provided to allow replication by others?

Yes

Reviewer Expertise:

Proteostasis, mitophagy C. elegans

I confirm that I have read this submission and believe that I have an appropriate level of expertise to confirm that it is of an acceptable scientific standard.

# Reviewer report: Reviewer response for version 1

**DOI:** 10.21956/openreseurope.15353.r28892

**Published:** 2022-04-29

**Authors:** Prashant Mishra

**Affiliations:** 1 UT Southwestern, Dallas, USA

Vozdek *et al.* report on the use of a mCherry:PDR1 reporter in C.elegans. PDR-1 is an ortholog of mammalian Parkin, which has been implicated in familial forms of Parkinson's disease, mitophagy, and organelle morphology. Here the authors make use of their reporter to identify Parkin interactors in C.elegans, as well as potential functions of Parkin in this model system.

 The authors find that their mCheery:PDR1 reporter largely localizes with lysosomal structures, and is not recruited to mitochondria under decoupling conditions (based on image analysis). Mass-spec immunoprecipitation and genetic screens identify a number of mitochondrial proteins as potential interactors and regulators of their PDR1 reporter. In addition, they find that their reporter alters processing of Synuclein inclusions and lysosomal morphology.

 Overall the data is convincing and technically sound. The relevance is limited, as the authors are studying an overexpressed and mCherry fused version of Parkin; thus, it is unclear if their findings are relevant to native/endogenous Parkin, particularly since their construct does not replicate key features of endogenous Parkin (including cytosolic localization and mitochondrial recruitment (Fig. 1)). Thus, although the work emphasizes the consequences and potential actions of Parkin overexpression in worms, my major recommendation would be to investigate if these results (interactors, effects on lysosomal dynamics) are properties of endogenous Parkin.

Is the study design appropriate and does the work have academic merit?

Partly

Is the work clearly and accurately presented and does it cite the current literature?

Partly

If applicable, is the statistical analysis and its interpretation appropriate?

I cannot comment. A qualified statistician is required.

Are all the source data underlying the results available to ensure full reproducibility?

Yes

Are the conclusions drawn adequately supported by the results?

Yes

Are sufficient details of methods and analysis provided to allow replication by others?

Yes

Reviewer Expertise:

Mitochondrial Disease; mitochondrial DNA

I confirm that I have read this submission and believe that I have an appropriate level of expertise to confirm that it is of an acceptable scientific standard.

# Reviewer report: Reviewer response for version 1

**DOI:** 10.21956/openreseurope.15353.r28891

**Published:** 2022-04-26

**Authors:** Anat Ben-Zvi

**Affiliations:** 1 Ben-Gurion University of the Negev, Beersheba, Israel

The manuscript "Caenorhabditis elegans Parkin: Regulators of its abundance and role in autophagy-lysosomal dynamics" by Vozdek *et al.* aims to identify new regulators of Parkin (pdr-1) in C. elegans. To this end, they establish a novel transgene encoding mCherry-tagged pdr-1. This transgene is enriched in the autophagy-lysosomal system and modulates mitochondrial morphology in the hypoderm. Using this transgene, the authors then perform forward and reverse genetic screens and protein-protein interactions to examine for modifiers of transgene abundance and localization. Finally, they examine the impact of this transgene on alpha-Synuclein inclusions. 

 The main concern I have is the possibility that the mCherry::PDR-1 construct is toxic, affecting the interpretation of the results. The authors themselves state in the discussion that "we cannot exclude the possibility that mCherry tag at the N-terminal autoinhibitory Ubl domain may alter its regulation, targeting and/or subcellular localization." The authors did not address this concern experimentally. While they showed that mCherry::PDR-1 altered mitochondria morphology (Fig 1A) compared to pdr-1 (mutant?) alone, I do not agree that the construct restored mitochondria morphology to that of WT. Moreover, known (and simple) functional assays examine pdr-1 function and could be used to determine the construct functionality—for example, the impact of pdr-1 on truncated mtDNA levels (see suggested ref below).

 The modifiers identified in the forward genetic screen reinforce this concern. Most are associated with RNA interference and silencing and point to the possibility that the expression of the mCherry::PDR-1 construct is activity silenced. In this regard, an important control will be to examine the impact of these mutations on pdr-1(+) expression by examining mRNA, or better yet, protein levels—alternatively, the effect of these mutants on other pdr-1 constructs. 

 These issues need to be addressed in revisions. 

 Minor comments: 

1. Several works on PDR-1 function in C. elegans are not referenced see the reference list for examples.
2. There is no statistical information in the manuscript. Specifically, Figs. 3B, 5B, and 6C.
3. It is not clear that pdr-1/parkin in the legend refers to pdr-1 mutant and which mutant.
4. What are "lost the dynamic movements in the hypodermal tissue," and how were they measured.

Is the study design appropriate and does the work have academic merit?

Partly

Is the work clearly and accurately presented and does it cite the current literature?

Partly

If applicable, is the statistical analysis and its interpretation appropriate?

No

Are all the source data underlying the results available to ensure full reproducibility?

Yes

Are the conclusions drawn adequately supported by the results?

Partly

Are sufficient details of methods and analysis provided to allow replication by others?

Yes

Reviewer Expertise:

proteostasis, mitophagy C. elegans

I confirm that I have read this submission and believe that I have an appropriate level of expertise to confirm that it is of an acceptable scientific standard, however I have significant reservations, as outlined above.

## References

[1] : Maintenance and propagation of a deleterious mitochondrial genome by the mitochondrial unfolded protein response. *Nature* .2016;533(7603) : 10.1038/nature17989 416-419 10.1038/nature17989 PMC487334227135930

[2] : Homeostatic Responses Regulate Selfish Mitochondrial Genome Dynamics in C. elegans. *Cell Metabolism* .2016;24(1) : 10.1016/j.cmet.2016.06.008 91-103 10.1016/j.cmet.2016.06.008 PMC528749627411011

[3] : Parkin modulates heteroplasmy of truncated mtDNA in Caenorhabditis elegans. *Mitochondrion* .2015;20: 10.1016/j.mito.2014.11.001 64-70 10.1016/j.mito.2014.11.001 25462019
